# Generation Mechanisms of Rotating Stall and Surge in Centrifugal Compressors

**DOI:** 10.1007/s10494-017-9877-z

**Published:** 2017-11-23

**Authors:** Elias Sundström, Bernhard Semlitsch, Mihai Mihăescu

**Affiliations:** 1 KTH - Mechanics, Competence Center for Gas Exchange (CCGEx), Osquars Backe 18. Royal Institute of Technology (KTH), Department of Mechanics, Stockholm, 10044 Sweden; 2 Whittle Laboratory - Department of Engineering, University of Cambridge, 1 JJ Thomson Avenue., Cambridge, CB3 0DY UK

**Keywords:** Rotating stall, Surge, Centrifugal compressor, LES

## Abstract

Flow instabilities such as Rotating Stall and Surge limit the operating range of centrifugal compressors at low mass-flow rates. Employing compressible Large Eddy Simulations (LES), their generation mechanisms are exposed. Toward low mass-flow rate operating conditions, flow reversal over the blade tips (generated by the back pressure) causes an inflection point of the inlet flow profile. There, a shear-layer induces vortical structures circulating at the compressor inlet. Traces of these flow structures are observed until far downstream in the radial diffuser. The tip leakage flow exhibits angular momentum imparted by the impeller, which deteriorates the incidence angles at the blade tips through an over imposed swirling component to the incoming flow. We show that the impeller is incapable to maintain constant efficiency at surge operating conditions due to the extreme alteration of the incidence angle. This induces unsteady flow momentum transfer downstream, which is reflected as compression wave at the compressor outlet traveling toward the impeller. There, the pressure oscillations govern the tip leakage flow and hence, the incidence angles at the impeller. When these individual self-exited processes occurs in-phase, a surge limit-cycle establishes.

## Introduction

The operating range of compressors is bounded by flow instabilities at low mass-flow rates; e.g. rotating stall and surge [1]. Centrifugal compressors are commonly employed in automotive turbochargers to increase the specific efficiency of internal combustion engines [2]. A wide compressor functionality range is essential to supply boost pressure at all engine operating conditions [3]. The understanding of the generation mechanisms leading to these flow instabilities would allow a more efficient flow control design, which yet puzzles researchers and engineers.

Rotating stall is a localized phenomenon restricted to the vicinity of the impeller, where stall cells propagate circumferentially across the blade passages. Different rotating stall appearances have been documented [1, 4] and control models have been developed [5]. Bousquet et al. [6] described the generation process in centrifugal compressors as a momentum balance between the incoming flow stream and the leakage flow at the impeller blade tip, inducing reversed flow. Rotating stall has been reported to be a precursor of surge [7, 8], while other researches [9] observed surge without traces of rotating stall.

Fink et al. [10] highlighted by investigating different machinery setups that surge is an insta- bility affecting and being characterized by the entire system. They described surge as a cyclic emptying and filling process of the volume between the downstream plenum and the impeller. Based on this description, they developed a low-order model, which could reproduce the system behavior. Although the general behavior of surge has been characterized, the flow dynamics around the impeller remained yet undisclosed.

The visualization of flow structures via experimental methods is challenged by the confinement of the geometry and the violent flow fluctuations at off-design operating conditions [11–13]. Nonetheless, Particle Image Velocimetry (PIV) measurements of the ingested flow upstream of the impeller have been performed by Guillou et al. [14], who reported strong swirling back flow at the outer perimeter at off design operating conditions. Semlitsch et al. [15, 16] could confirm by studying the same ported shroud compressor numerically that this reversed flow originates from the tip leakage and back flow jets generated via the ported shroud. Semlitsch and Mihăescu [17] suggested that the increased tip leakage swirling the inflow toward off design operating conditions degenerates the incidence flow angles such that the rotor blades might be incapable to maintain a constant back pressure and cause surging.

This emphasizes the practicality of unsteady computational methods such as LES for the investigation of flow structures in centrifugal compressors. Further, the potential of stability analysis via numerical methods to predict the onset of stall was shown by Sun et al. [18], Stein et al. [19] and Sundström et al. [20]. Vortical flow structures circulating at the impeller inlet have been visualized by Sundström et al. [21] and Broatch et al. [22]. These, could be associated to rotating stall.

The aim of the present work is to understand the link of rotating stall and surge in centrifugal compressors and the generation of these phenomena. Therefore, we perform compressible LES calculations to investigate the flow structures at different operating points. We show that the vortical flow structures roll off the inflection point radius of the inlet profile and are therefore, linked to the tip leakage back-flow. The modes associated with these flow structures are analyzed using the modal decomposition techniques and Power Spectral Density (PSD). Further, we present the flow field at surge with a statistical analysis of the incidence flow angles, the flow field around the blades and the transferred flow momentum in order to reveal the generation mechanism of surge.

## Geometry and Numerical Methodology

**Fig. 1:**
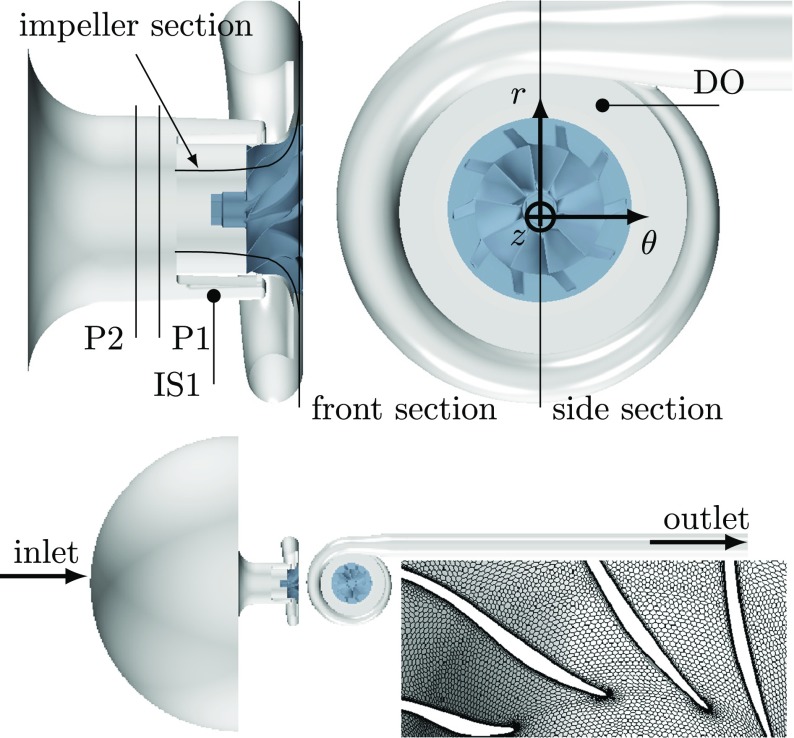
Side and front views of the centrifugal compressor CAD geometry. Locations of the pressure sensors D0 and IS1 are indicated as well as the location of planes used during data post-processing. The IS1 point is located in the middle of the ported shroud cavity, oriented 127^∘^clockwise from the vertical and 0.22*D*_2_from the diffuser back wall. The impeller section intersects the impeller region at 50% blade span. The P1 and P2 planes intersect the inlet duct upstream of the impeller eye. These planes are located 0.85*D*_2_and 0.76*D*_2_, respectively, upstream from the diffuser back wall. The bottom subfigure depicts inlet and outlet boundaries of the computational domain. The inset at the bottom right shows a zoomed in view of the blade passage grid resolution at 50% blade span

The investigated centrifugal compressor is presented in Fig. 1, where the impeller contains ten main blades with an exducer diameter *D*_2_ = 88 mm, and the vaneless diffuser has an area ratio of 0.57. The compressor is equipped with a ported shroud, which is supported by four asymmetrically arranged ribs. The main objective of the ported shroud is to extend the stable operating range of the compressor map (see e.g. Yang et al. [23]).

Four different operating conditions are considered at a constant speedline of 64000 rpm to investigate the evolution of flow instabilities developing toward the low mass-flow rate limit. Details to the operating conditions are provided in Table 1. Case D corresponds to a stable operating condition, whereas the other cases are chosen in the operating regimes of rotating stall and surge.

**Table 1:** Imposed inlet and outlet boundary conditions

Case	$\dot {m}$ [kg/s]	*p*_*in*_ [kPa]	*T*_*in*_ [K]	*T*_0,*exit*_ [K]
A	0.050	101.325	300	365
B	0.085	101.325	300	365
C	0.105	101.325	300	365
D	0.280	101.325	300	344

An idealized installation is considered to mimic the experimental setup at the University of Cincinnati [14]. Air is entrained from quiescent, standard ambient conditions via a bell mouth inlet. The hemispherical shaped inlet is located three diffuser diameters upstream from the bell mouth entrance to the compressor, where a non-reflective boundary condition treatment is applied. The region close to the compressor can be considered as a compact acoustic source field, which radiates acoustical waves in all directions toward the acoustic farfield at the speed of sound. To prevent such waves from begin reflected at the inlet boundary, i.e. back toward the compressor, the freestream velocity on the inlet cell face is corrected. The boundary normal velocity component on the cell face is obtained from characteristics, which involves extrapolation from the neighboring cell center velocity. A more detailed explanation of the non-reflecting boundary treatment can be found in the work by Giles [24].

Correspondingly to the experimental setup, the compressor outlet duct is ten diffuser diameters long. At the outlet, the mass-flow is fixed and so is the total temperature.^1^ Thus, the outlet pressure is allowed to float about the specific target pressure according to the experimental conditions. The walls are considered as adiabatic^2^ with non-slip boundary conditions.

The finite-volume compressible solver within STAR-CCM+ is utilized for the simulation of the flow. It is characterized by the integral form of the conservation equations of mass, momentum, and energy. The air flow is assumed to behave as an ideal gas, where the dynamic viscosity and thermal conductivity are assumed to be functions of the local temperature only. An implicit LES approach is adopted to reproduce the unsteady flow in the compressor.

An implicit second-order upwind scheme is used for time marching. For the calculation of the convective and diffusion gradients, an implicit, bounded central third-order scheme is employed. The governing system of equations is solved as a coupled system with Gauss-Seidel relaxation in an algebraic multigrid approach. To handle the impeller rotation, the domain is divided into stationary and rotating regions, which are interfaced by the sliding mesh technique requiring information interpolation at each time step. A solid body rotation at an angular velocity of the impeller is applied to revolve the grid about the compressor axis. The computational domain is discretized by a fine unstructured polyhedral grid consisting of approximately nine million finite volumes. A low-Reynolds number wall-resolved grid with *y* ^+^ = 1distribution on average is used. This is obtained with 10 prismatic cell layers, a total wall prism layer height of 0.5 mm and using a geometric stretching factor of 1.5.

The simulations have been computed for 0.2 s to converge the spectral averages corresponding to 200 rotor revolutions. More specifications of the numerical methodology have been documented in previous publications, see i.e. Sundström et al. [20, 21], where also a grid sensitivity study and validation with experimental data were performed. Therefore, the presently employed approach can be considered as a reliable technique to capture the flow dynamics in the compressor.

## Results

**Fig. 2:**
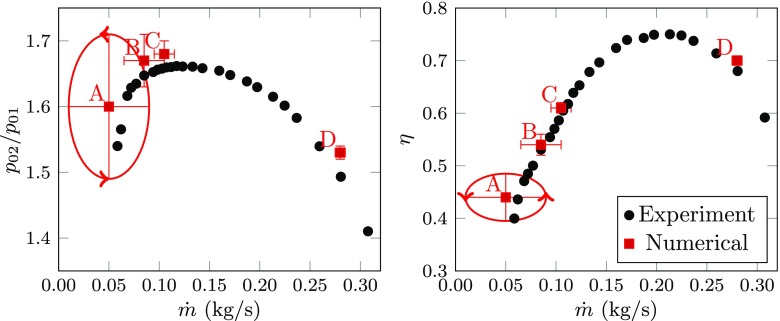
The performance map illustrates the location of the chosen operating conditions. The variation of the total pressure ratio and isentropic efficiency during operation is illustrated with horizontal and vertical bars, respectively. A limit cycle is established for Case A, which is depicted with a circle and two arrows in order to show the phase direction. Experimental data [26] is added to guide the reader

The operating conditions under investigation are characterized by the fluctuations of the global performance parameters, as shown in Fig. 2.^3^ Small variations to the mean value can be observed for stable operating condition, while the perturbations are enhanced toward low mass-flow rate operating conditions. These are the fundamental properties of flow instabilities manifesting on the positive slope of the characteristics. With surge, the performance parameters oscillate and form a limit cycle,^4^ where the amplitude and frequency are governed by the entire system [25].

**Fig. 3:**
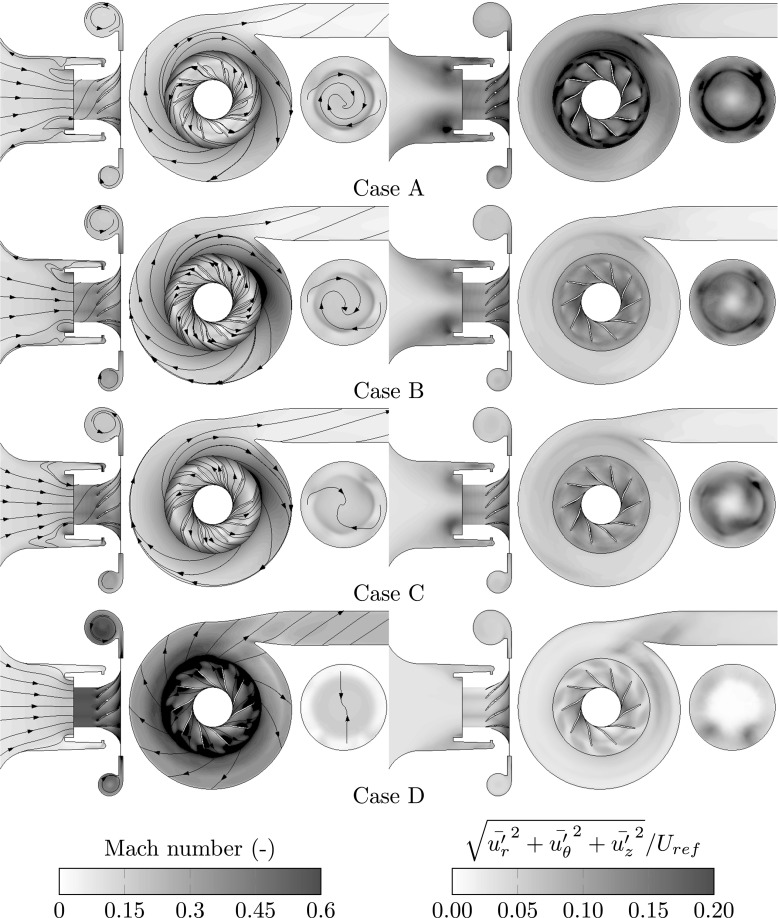
Flow-field characterization at the four investigated operating points in terms of time-averaged Mach number and turbulent kinetic energy. Streamlines of the velocity field are overlapped to indicate the flow direction. For the location of the post-processing planes see Fig. 1. Here, the P1 plane is offset to the right relative the front plane

The observed attributes at off-design operating conditions express themselves also in the flow-field, which is shown in Fig. 3. The flow velocities are lower at low mass-flow rates when the static back pressure is higher. This causes a higher residence time of the flow, manifested in the form of circulating the flow in the radial diffuser with intensified turbulent fluctuations. The turbulent kinetic energy levels increase significantly the further the mass-flow rate is reduced. The level is specifically increased at the interface between the diffuser and the volute, subject to flow unsteadiness at the trailing edge, which is similar with observations made by Lennemann and Howard [27].

**Fig. 4:**
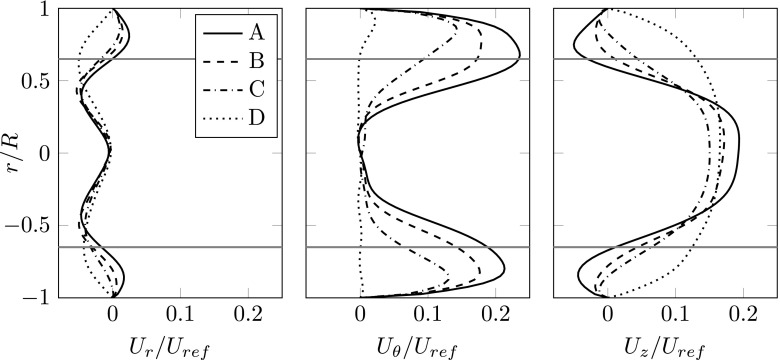
Time averaged velocity profile components in radial, tangential and axial directions. All profiles are evaluated along a vertical line at the plane P2. The blade tip speed, *U*_*ref*_, is used for normalization. The gray horizontal lines indicates the circumferential radius at which the rotating-stall vortical structures are circulating in the P1 plane. This location is close to the inflection point

The higher back pressure pushes the flow through the blade tip gap toward the shroud inlet. This back flow, which increases the further the mass-flow rate is reduced, is quantified in Fig. 4. Due to the rotation of the rotor, the back flow exhibits inherently a swirling motion in the same rotational direction as the wheel. With the mixing process of the back flow and the freshly entrained air, the entire flow upstream of the impeller spins around the impeller axis. The high turbulent kinetic energy levels (Fig. 3) indicate the mixing of the back flow with the entrained stream upstream of the impeller, in the shroud entrance.

Toward surge operating conditions, the formation of an annular separated, radial outwards and inwards swirling flow can be clearly observed in the P1 plane shown in Fig. 3 (in terms of streamlines) and on the P2 plane in Fig. 4. The flow profiles on the P2 plane (shown in Fig. 4) reveal a significant amount of back flow, which acts as a blockage for the freshly entrained air. Therefore, the inflow is funneled causing the radial flow profile, while the back flow spreads outwards due to the centrifugal forces. Both the radial and axial velocity profiles yield an inflection point, a possible onset mechanism for shear-layer instability. Coinciding with this shear-layer interface, an annular increased region of turbulence kinetic energy can be observed in the P1 plane shown in Fig. 3.

**Fig. 5:**
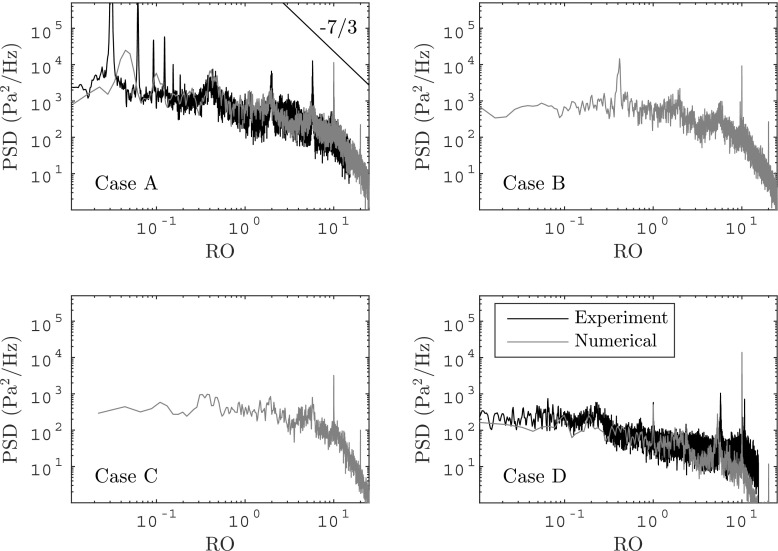
The Power Spectral Density (PSD) at probe location IS1 (see Fig. 1 for location orientation) is shown for all investigated operating conditions, respectively. Experimental data [26] is added to guide the reader. Comparison for Case B and Case C was omitted because the experimental data exhibits non-investigated features, who’s origin is undetermined and is not visible in the numerical data. The frequency axis is normalized with the angular velocity of the impeller shaft, represented as the Rotating Order (RO). A RO= 1 corresponds to one full rotation

The spectra shown in Fig. 5 exhibit tonalities on top of a broadband distributed fluctuation character. These can be related to the angular velocity of the impeller shaft (RO), blade passing frequency (BPF), rotating stall (RS) and surge (S). There are also frequencies related to duct resonance, where multiples (2 and 5) of the rotating order are amplified. For the stable, near design operating condition (Case D) only the RO= 1, RO= 5, and the blade passing frequency RO= 10 with its higher harmonic RO= 20 can be observed, respectively. Additionally, a tonality in the range of approximately 30 − 50*%* of the rotating order unity for all low mass-flow rate operating conditions is observed.^5^ Due to the influence of the dominant surge oscillation at a lower frequency, the rotating stall establishes as a narrowband amplification over a finite frequency range for Case A. Further, another clearly distinct peak at RO= 0.04 (43 Hz) can be observed, including one harmonic at RO= 0.08. Similarly, this peak also spans a range of frequencies, which is due to the period variation over time. It suggests a complex situation with multiple period doubling until the signal may eventually come back to the same initial starting point. In between the tonalities, the spectrum is broadband. Thus the signal in those frequency ranges is stochastic with no apparent coherent flow structure.

### Flow Field Decomposition Revealing Rotating Stall and Surge

**Fig. 6:**
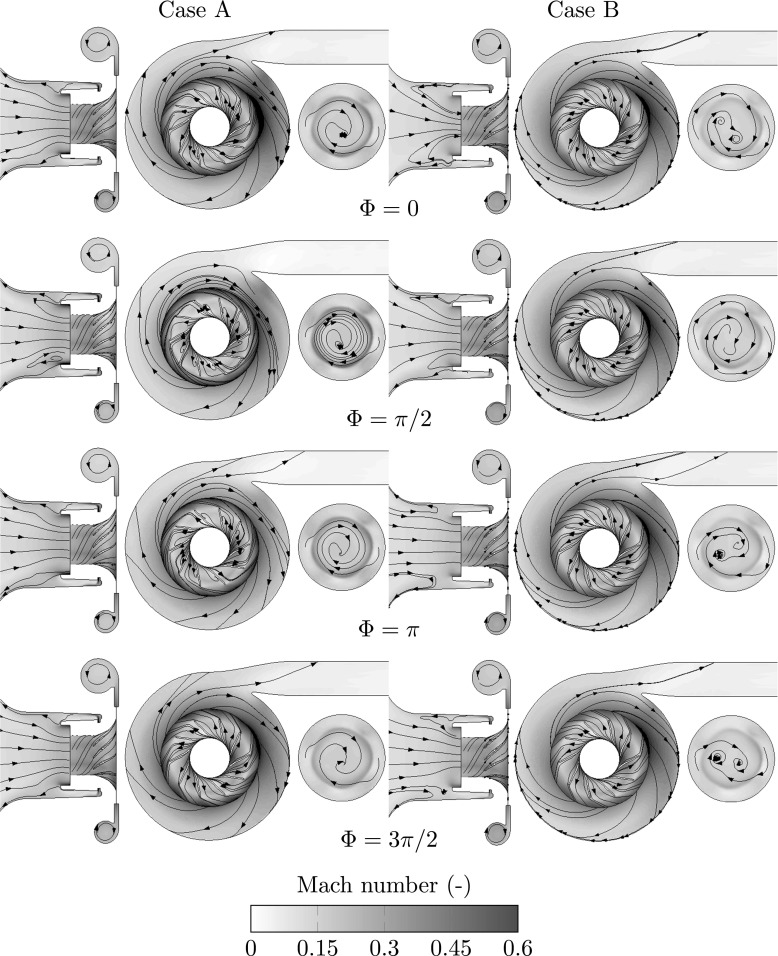
Velocity streamlines and Mach number evolution of the surge and rotating stall modes are shown. Case A (left column) is phase averaged at the surge frequency tonality and Case B (right column) is phase averaged at the rotating stall frequency tonality. For location of post-processing planes see Fig. 1. Here, the P1 plane is offset to the right of the front plane

**Fig. 7:**
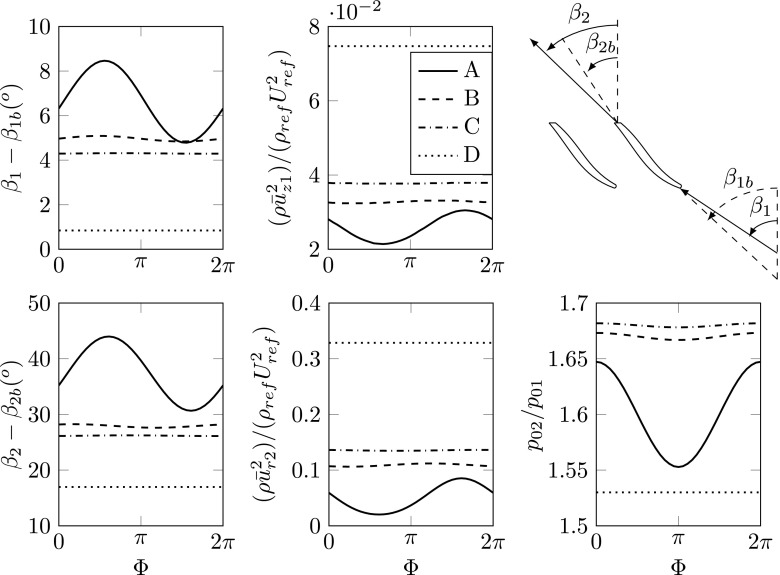
Quantities characterizing the impeller performance are presented as function of the cycle phase at the dominant frequency for the particular case, i.e. Case A, surge; Case B & C, rotating stall; Case D, time-averaged. The quantities, i.e. flow incidence and discharge angles, upstream and downstream flow momentum and total pressure ratio, are circumferentially averaged at 50*%* blade span. The inducer blade angle is *β*_1*b*_ = 60^∘^and the blade back sweep angle is *β*_2*b*_ = 40^∘^, defined relative the meridional, see sketch in the upper right corner

Modal decomposition of the sampled simulation data was performed to analyze the instabilities associated with the flow phenomena. The flow field is therefore reconstructed in terms of mean and solely the modal oscillation at the particular frequency corresponding to rotating stall (Case B) and surge (Case A). Hence, the modal decomposition is utilized to filter out the frequency of interest from the unsteady flow. The reconstructed sequence as function of its phase is shown in Fig. 6, while quantitative analysis of the impeller work is presented in Fig. 7.

At the rotating stall frequency of approximately 30 − 50*%* of the rotating order unity (Case B), the most prominent feature is a co-rotating vortex pair being rolled off at the inner side of the shear layer formed by the back flow in the compressor inlet. Its evolution is shown in Fig. 6 in the P1 plane. Following streamlines of the phase angle sequence, it is noteworthy that the vortex pair propagates only a half revolution per rotating stall cycle. Inspecting the streamlines close to the volute tongue over a rotating stall cycle in Fig. 6, it can be observed that the flow intensely recirculates into the volute (between Φ = 3*π*/2 and 0) while the flow discharges into the compressor exit pipe at other times of the cycle (between Φ = *π*/2and *π*). Hence, the forming shear layer under the volute tongue [15] is excited by the perturbations generated via the stall cells. Further, the strength of the vortex changes, which depends on its circumferential location. This is noted by the coiling up of the streamlines around the vortex cores. It is influenced by the non-axisymmetric pressure distribution in the radial diffuser at low mass flow rate operating conditions [28]. It is also influenced by the back flow distribution [29]. At Φ = 3*π*/2, the two streamlines on the P1 plane, seeded outside of the shear-layer, diverge radially outwards due to the centrifugal force. Streamlines seeded inside of the shear-layer eventually end up in the co-rotating vortex pair.

The manifestation of this flow instability, i.e. rotating stall, evolves similarly as described by Alekseenko et al. [30] who analyzed a simplified scenario where helical vortices where found in a swirling flow. The radial gradient of the radial velocity component (shown in Fig. 4) generates the instability causing the vortices. The rotation of the co-rotating vortices around the impeller axis occurs due to radial gradient of the circumferential velocity component.

The essential flow feature of surge is the global response of the compression system, which is characterized by flow recirculation causing the emptying and filling process. By a phase angle of 3*π*/2shown in Fig. 6 for Case A, the compressor undergoes the filling phase of surge. The flow incidence angles at the leading edge of the impeller reach a minimum (as shown in Fig. 7), which coincide with similar inflow angles as observed for Case B. Therefore, the flow momentum transferred downstream reaches high values and the streamlines shown in Fig. 6, especially in the blade passages, indicate an efficient mass flow transport downstream. The high flow momentum transported downstream causes the pressure to build up in the compressor discharge pipe, which reaches a maximum at a phase angle of 2*π* (phase shift of *π*/2compared to the momentum). The phase lag of the pressure behind the moment is evidently shown in Fig. 7. By the phase angle 0 in the surge cycle, more flow is pushed by the high pressure in the discharge pipe and recirculated in the volute underneath the tongue. By Φ = *π*/2the compressor undergoes the emptying phase, where the pressure at the discharge pipe decays rapidly. The streamlines at the inducer reverse mid-way through the blade passages with large flow separation zones, resulting in a high flow discharge angle and low flow momentum transport, as shown in Fig. 7. The two seeded streamlines on the P1 plane are subsequently being trapped with an extremely long residence time and forms multiple turns around the center axis. The impeller is not capable to retain its efficiency. Again, the pressure lags *π*/2behind the low flow momentum indicating a resonance phenomenon.

**Fig. 8:**
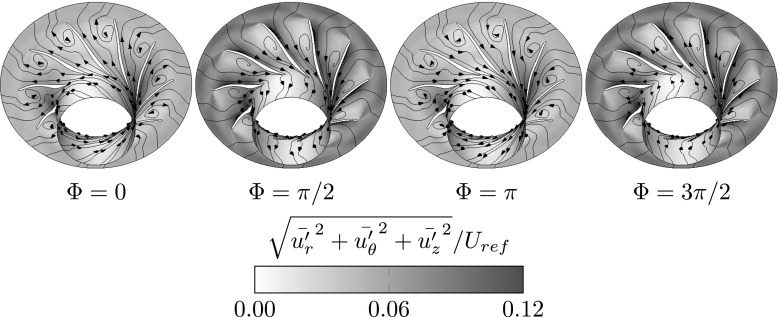
Phase evolution of the modal flow perturbation within the blade’s passages at the surge frequency (Case A). Flow streamlines on top of normalized turbulence kinetic energy. The data is circumferentially averaged. Between Φ = 3*π*/2and Φ = 0the incoming flow streamlines are directed downstream. From Φ = *π*/2to Φ = *π* the streamlines depict complete flow reversal for an averaged blade passage

As explained in the previous paragraph, the crucial factor leading to surge is the flow incidence angle at the impeller blade tip, with impact on the rotor efficiency to push fluid downstream. Figure 8 presents the modal flow perturbation at the surge frequency (case A), in terms of flow streamlines overlapped on the turbulence kinetic energy data. The data, circumferentially averaged, reveals the variations of the incoming flow incidence angle, see e.g. cycle phase Φ = 3*π*/2 as compared with cycle phase Φ = 0. Moreover, the streamlines indicate large regions of flow separation off the blade surfaces toward the impeller discharge. This complements Fig. 7, which shows at surge (Case A) that only very little flow momentum is transferred downstream of the impeller. Thus, only a proportion of the impeller cord imparts flow momentum.

By the phase angle Φ = 0the streamlines close to the blade pressure side and in the middle of the blade passage depict downstream directed flow and the turbulence kinetic energy level is at a minimum, as shown in Fig. 8. Close to the blade suction side, the streamlines describe a circular motion (located toward the rear of the blade). They are spiraling outwards and finally are discharged downstream, into the diffuser. Qualitatively, this feature is typical for an emerging boundary layer separation. By Φ = *π*/2 all seeded streamlines are now going in the upstream direction depicting reversed flow of the surge mode and the turbulence kinetic energy distribution shows a strong gradient between the pressure and suction sides of the blade. The streamline seeded close to the blade suction side initially describes an outward spiraling motion before its being discharged upstream. Since the seeded streamlines are completely reversed, the impeller is not producing any notable downstream momentum (see also Fig. 7 at Φ = *π*/2). Toward phase angle Φ = *π* in Fig. 8, the turbulence levels have now dropped. The seeded streamlines still depict flow reversal, but the angle at which the flow moves upstream from the impeller is almost parallel with the impeller’s inlet plane. The compressor is gradually recovering from a minimum pressure level of the mode cycle (see also Fig. 7). By cycle phase of Φ = 3*π*/2all streamlines show downstream oriented flow, apart from the streamline seeded close to the blade suction side. This correlates with flow incidence and discharge angles reaching their minimum levels, but also to that the upstream and downstream flow momentum are at their maximum levels. From Φ = 3*π*/2 to Φ = 2*π* the flow mode perturbation will gradually go back to the phase evolution stated at Φ = 0, and the surge limit cycle will repeat.

## Conclusions

The unsteady compressible flow approaching surge operating conditions in a centrifugal compressor has been analyzed using LES. The numerical setup and the boundary conditions were chosen to replicate the cold gas-stand experiment at the University of Cincinnati. Therefore, validation of the numerical predictions with experimental data was possible and documented by Sundström et al. [20, 21].

Focusing on flow instabilities, the flow field evolution from design to off-design conditions was assessed. Tonalities in the pressure spectra corresponding to rotating stall and surge have been shown for particular low mass-flow rate operating conditions. Flow modal decomposition analysis was employed to investigate the phenomena causing rotating stall and surge.

The observation of back flow emergence toward off-design operating conditions has been documented by many investigations, both numerically [31] and experimentally [26]. This back flow, streaming over the blade tips, is generated by the increased back pressures at low mass-flow rate conditions. We showed that flow incidence angles were altered by the back flow swirl, which lead to a reduction of entrained flow momentum and hence rotor efficiency. Surge was shown to be an extreme case, where the flow incidence angle and hence the downstream flow momentum transfer oscillate in a feedback loop with the build-up of back pressure in the discharge pipe causing a limit cycle.

In a surge limit cycle it was shown that the pressure ratio and the entrained flow momentum are phase shifted 90^∘^. Thus, at peak pressure ratio, the flow momentum is 90^∘^from reaching its minimum. The incidence angle is seen to be in phase with the momentum. When the incidence angle and the momentum reach their maximum and minimum, respectively, the pressure ratio has reduced significantly and is well on its way toward the minimum value. But since the back pressure drops it allows the flow momentum to increase and the impeller gradually recovers, and starts to produce gradually more downstream radial momentum. However, it only increases until some critical pressure ratio. Beyond this limit the momentum starts to drop again, and the surge limit cycle repeats.

At all low mass-flow rate operating conditions, the back flow over the blade tips interacts with the freshly entrained stream in the shroud entrance region, generating a strong shear-layer. In the presence and energized by the swirling back-flow the shear-layer can give rise to helical vortex formation circulating in the impeller inlet. The tonal signature of this flow instability can be traced far downstream into the radial diffuser and the volute. The asymmetric pressure distribution in the volute at off-design operating conditions results in an amplification of vortex strength toward the volute tongue. Therefore, the induced pressure oscillations are especially notable as modulation of the shear-layer under the volute tongue. Further, the perturbations induced at the impeller were shown to affect the achieved boost pressure ratio.

It was shown that the back flow at the impeller eye, manifesting due to tip-leakage, is essential for the occurrence of both phenomena, i.e. rotating stall and surge. However, the strength of the generated shear-layer in the shroud entrance governs rotating stall, whereas the induced swirl is decisive for surge. The shear-layer strength was also shown to be affected by the swirling motion of the back-flow, as quantified with the Reynolds stresses upstream of the impeller eye.

It was found that the flow separates near the shroud wall with flow reversal going in the upstream direction, i.e. for operating conditions close to the surge line. This flow reversal induces a strong shear-layer interface. Seeded streamlines on the inner side of the interface were shown to curl up into two vortex pairs. Moreover, since a strong swirl component is present under surge condition, these vortices are seen to co-rotate in the same direction as the impeller rotation. For Case B, i.e. not in the deep surge condition, this feature is seen to be relatively distinct in the point spectrum, which means that one rotating stall period is similar with the next following one. For Case A, the rotating stall feature is seen to be more spread out over a larger frequency range. Thus, in presence of strong swirl the strength and frequency of co-rotating vortex pair are modulated. By means of seeded streamlines, it was qualitatively shown that the co-rotating vortex pair gains strength, when one of the vortices is aligned with the volute tongue.

## Nomenclature

ᅟ: ᅟ

## Latin symbols

$\dot m$: mass-flow rate [kg/s]
*M**a*: Mach number [-]
*p*: pressure [Pa]
*T*: temperature [K]
*U*: mean velocity [m/s]
*D*_2_: exducer diameter [m]

## Greek symbols

*β*: angle [^*o*^]
*ρ*: density [kg/m^3^]
*γ*: ratio of specific heats [-]
*η*: isentropic efficiency $(\frac {p_{02}}{p_{01}}^{\frac {\gamma -1}{\gamma }}-1)/(\frac {T_{02}}{T_{01}}-1)$ [-]
Φ: phase angle [radian]

## Abbreviations

BPF: Blade Passing Frequency
PSD: Power Spectral Density
LES: Large Eddy Simulation
PIV: Particle Image Velocimetry
RO: Rotating Order. Normalized angular velocity

## Subscript

0: total quantity
1: upstream of the impeller
2: downstream of the impeller
*r**e**f*: variable is referred to reference value
*b*: blade
*r*, *𝜃*, *z*: radial, tangential and axial coordinates

## Footnotes

1 The back pressure and thus the mass flow is regulated via a valve in the experiments [14], which has been modeled by a mass flow rate boundary condition in the simulations due to the lack of the valve geometry information. The valve exhibits different impedance characteristics delaying the pressure reflection as compared to the numerical boundary condition. Therefore, surge occurs at a lower frequency in the experiments as in the simulations. Nonetheless, it can be observed that the employed numerical method captures all other tonalities.

2 This represents another difference to the experimental setup, where the compressor is driven by the turbine.

3 The disagreement can be explained by the differences in the experimental and numerical setup, as mentioned in the section “Geometry and Numerical Methodology”.

4 The limit cycle is obtained from the raw monitored system variables for Case A, i.e. $p_{02}/p_{01}(t), \eta (t), \dot {m}(t)$. These signals are first low-pass filtered and then approximated with a Fourier curve fit of 1st order. This yields the surge frequency, the phase angle, and their respective amplitudes. Since the phase angle is 90^*o*^, an ellipse shape in the phase space diagram is obtained with a semi-major axis (*p*_02_/*p*_01_ amplitude) and semi-minor axis ($\dot {m}$ amplitude).

5 Rotating stall was observed at approximately 480 Hz, 425 Hz and 320 Hz for the Cases A-C, respectively.
